# Sub-clustering in skeletal class III malocclusion phenotypes via principal component analysis in a southern European population

**DOI:** 10.1038/s41598-020-74488-w

**Published:** 2020-10-21

**Authors:** L. de Frutos-Valle, C. Martin, J. A. Alarcón, J. C. Palma-Fernández, R. Ortega, A. Iglesias-Linares

**Affiliations:** 1 Section of Orthodontics, Faculty of Odontology, Complutense University, Madrid, Spain; 2 Craniofacial Biology Research Group, BIOCRAN, Complutense University, Plaza Ramón y Cajal, s/n, 28040 Madrid, Spain; 3 Section of Orthodontics, Faculty of Odontology, University of Granada, Campus Universitario de Cartuja, Granada, Spain; 4 Faculty of Odontology, Complutense University, Madrid, Spain

**Keywords:** Orthodontics, Malocclusion

## Abstract

The main aim of this study was to generate an adequate sub-phenotypic clustering model of class III skeletal malocclusion in an adult population of southern European origin. The study design was conducted in two phases, a preliminary cross-sectional study and a subsequent discriminatory evaluation by main component and cluster analysis to identify differentiated skeletal sub-groups with differentiated phenotypic characteristics. Radiometric data from 699 adult patients of southern European origin were analyzed in 212 selected subjects affected by class III skeletal malocclusion. The varimax rotation was used with Kaiser normalization, to prevent variables with more explanatory capacity from affecting the rotation. A total of 21,624 radiographic measurements were obtained as part of the cluster model generation, using a total set of 55 skeletal variables for the subsequent analysis of the major component and cluster analyses. Ten main axes were generated representing 92.7% of the total variation. Three main components represented 58.5%, with particular sagittal and vertical variables acting as major descriptors. Post hoc phenotypic clustering retrieved six clusters: C1:9.9%, C2:18.9%, C3:33%, C4:3.77%, C5:16%, and C6:16%. In conclusion, phenotypic variation was found in the southern European skeletal class III population, demonstrating the existence of phenotypic variations between identified clusters in different ethnic groups.

## Introduction

Class III malocclusion has been commonly described as having a retruded or hypoplastic upper maxilla, a prognathic or hyperplastic mandible, or a combination of both^1^. These shared characteristics in skeletal class III malocclusion vaguely define the different sub-phenotypes clearly identified in recent studies^2–8^.

The current diagnosis of skeletal radiographic analysis includes multivariate techniques that allow the phenotypic characterization of different groups of class III skeletal malocclusions. Principal component analysis (PCA) enables the large number of variables used in cephalometric analysis to be reduced to fewer components, thus grouping the variables of greatest interaction on each axis^9^. This has made it possible to create phenotypic groups (clusters) for this specific malocclusion using cluster analysis to ascertain the least dispersion in each group and concomitantly achieve the greatest difference between each of the groups found^10^.

The number of distinguishable skeletal class III sub-phenotypes varies substantially between different studies and populations described in the literature: from seven different sub-phenotypes in Korean adults^2^ to three^3,6,7^ clinically distinguishable clusters in growing Caucasian patients; and four^8^, five^5^, and 14^4^ skeletal class III clusters (C) have also been obtained via cluster analysis (CA) and described in the literature. These studies clearly show great variations within the same skeletal malocclusion and can be classified in different phenotypic groups with specific characteristics that differ from one ethnic group to another, and even within the same ethnic group^2–8,11^. The aforementioned studies have extensively described different numbers of class III malocclusion sub-phenotypes by taking into consideration skeletal and dental, dentoalveolar, and soft tissue measurements in different ethnicities and age groups^11^. The use of skeletal cephalometric measurements alone to perform these analyses may ensure that only skeletal clusters are identified, thus avoiding the interaction of dental components and soft tissue variations that could mask an accurate skeletal perception^12^. The main purpose of the present investigation therefore is to generate an appropriate sub-phenotypic grouping of skeletal class III malocclusion in a broad adult population of southern European origin.

## Materials and methods

### Study design and ethics statement

This report consists of a two-phase study comprising a preliminary cross-sectional study and a subsequent discriminatory assessment via principal component and cluster analysis that allowed the identification of skeletally differentiated sub-groups with specific phenotypical characteristics. First, we aimed to identify the radiographic characteristics of a skeletal class III sample. The subjects were then classified into clusters according to these characteristics to establish a discriminatory analysis that enabled the identification of radiographically differentiated class III sub-phenotypes.

This study was conducted in accordance with the principles of the Declaration of Helsinki^13^ safeguarding the rights and interests of those involved in research. The study protocol was approved by the Institutional Ethics Committee (CE) of the Complutense University of Madrid (Clinical Research Ethics Committee of the San Carlos Clinical Hospital of Madrid, reference 17/063).

### Study population, eligibility criteria, and subject inclusion process

The study population comprised 699 patients of southern European ethnic origin from Spain seeking orthodontic treatment. Eligible subjects were recruited from patients affected by skeletal class III malocclusion attending the Orthodontic Postgraduate Program at the Complutense University of Madrid and other private dental practices nearby. All the participants had been previously diagnosed with class III malocclusion by their doctors at each of these centers. All subjects were of southern European origin, had practically completed their growth as assessed by cervical vertebral maturation stage (CVMS)^14^ and given informed consent to participate in the research.

Final selection for inclusion was made after compliance with all other specific criteria (Supplementary Table 1). Of the initial 699 participants, a final selection of 212 adult skeletal class III participants was made (97 men and 115 women; mean age for men was 28 ± 10.66 and 29 ± 10.12 for women) for cluster generation.

Sample size estimation was based on information available from previous studies^5^. The sample size was calculated with a confidence level (1 − α) of 95%, statistical power of 90%, precision (d) of 0.30, and variance (S2) of the quantitative variable of reference group of 0.69. This calculation determined a total sample of 131 individuals. The sample size was then adjusted for losses; the expected proportion of losses (R) was fixed at 15%, and a sample size of 154 subjects was finally estimated.

### Clinical and diagnostic records

Pre-treatment lateral cephalometric radiographs of every patient were obtained prior to orthodontic treatment (Orthoceph by Siemens, Sirona Orthophos plus DS Ceph, Gendex Orthoralix). All patients were placed 10 cm away from the radiographic plate. All of the lateral radiographs were imported into Dolphin Imaging software (11.0, Dolphin Imaging & Management Solutions, Chatsworth, CA, USA) and calibrated and standardized with a 10 mm digital ruler. Chronological age was recorded and CVMS was determined^14^ in all patients to verify that they had no remaining significant craniofacial growth. In addition, patient gender and ethnic origin were noted as part of the required criteria for inclusion in the present research.

### Radiological landmarks, measurements, and axis model generation

A single experienced operator (L.F.V.) measured the craniofacial landmarks and cephalometric measurements via the radiometric software used in this study to avoid additional potential errors. The examiner underwent a calibration period prior to initiation of this research using a subset of duplicated radiological landmark positioning and measurement assessment.

Overall, 102 cephalometric measurements and 67 radiological landmarks were located in each patient (see Supplementary Table 2 for full description). Of these landmarks, 29 were on pure skeletal structures, 12 were on dental positions, 15 were in soft tissue structures, and the remaining 11 were related to airway structures. A total of 21,624 radiographic measurements were obtained as part of the cluster model generation. Each patient was examined and described via 102 radiographic variables (Table 1): 55 were skeletal measurements, 25 were dental parameters, and 22 were soft tissue (15) and airway (7) parameters. Among the skeletal parameters, 23 were angular measurements, 24 were distances, and 8 were proportional measurements (Table 1).

**Table 1:** Skeletal and supplementary craniometric variables.

Skeletal		Dental	Soft tissue	Air way
**Skeletal angular FH-SN (°)**	**Skeletal linear**	**Dental angular**	**Soft tissue angular**	**Air way angular OPT-NS (°)**
SNA (°)	Anterior cranial base (SN) (mm) Anterior face height (NMe) (mm) Upper face height (N-ANS) (mm) Lower face height (ANS-Me) (mm) Posterior cranial base (S-Ar) (mm) Posterior face height (SGo) (mm) Ramus height (Ar-Go) (mm)	Interincisal angle (U1**-**L1) (°) U1**-**NA (°)	NLA (Nasal angle) (°)	
SNB (°)	Co-Go (mm)	U1**-**SN (°)	Facial convexity (G'-Sn-Pg') (°) H-angle (Pg'UL-Pg'N') (°)	
ANB (°)	Convexity (A-NPg) (mm)	U1**-**Palatal Plane (°) U1**-**FH (°)		
SND (°)	Maxillary skeletal (A-N Perp) (mm) Midface length (Co-A) (mm)	L1**-**NB (°)		
Y-axis (SGn-SN) (°) SN**-**GoGn (°)	Ar**-**A (mm)	L1 to A-Pg (°) L1**-**FH (°)	**Soft tissue linear**	**Air way linear**
Cranio-Mx base/SN-Palatal plane (°) Occ plane to SN (°)	Maxillary length (ANS-PNS) (mm) Pg**-**NB (mm)	IMPA (L1-MP) (°)	Upper lip**-**S line (mm) Upper lip**-**VRP (mm)	Lower airway: Oro-pharyngeal Upper airway: Naso-pharyngeal Anterior nasal cavity height (mm) Posterior nasal cavity height (mm) H**-**PP (ANS-PNS) (mm)
Occ plane to FH (°)	Mand. skeletal (Pg-N Perp) (mm) Mandibular body length (Go-Gn) (mm) Length of mand base (Go-Pg) (mm) Mandibular length (Co-Gn) (mm)	L6 long axis**-**MP (°)	STissue N Vert (N Perp) to upper lip (mm) Lower lip**-**S line (mm)	PNS to basion (mm)
Facial axis-Ricketts (NBa-PtGn)(°) FMA (MP-FH) (°)	Co-B1 total mand (mm) Ar**-**Gn (mm)		Lower lip to E-plane (mm) Lower lip**-**VRP (mm)	
Lower face height (ANS-Xi-Pm)(°)	Basal width (mm)		STissue N Vert (N Perp) to lower lip (mm) STissue N Vert (N Perp) to ST Pogonion (mm) Sn'-Me' (mm)	
Facial angle (FH-NPg) (°) N-A-Pg (°)	Mx/Md diff (Co-Gn**-**Co-A)(mm) Wits (FOP) (mm)			
Facial taper (°)	Wits appraisal (mm)			
Gonial/jaw angle (Ar-Go-Me) (°) Upper gonial angle (Ar-Go-N) (°°) Lower gonial angle (Na-Go-Me) (°) Articular angle (°)				
Saddle/Sella Angle (SN-Ar) (°) Superior angle SN-AB (°)				
Rp-FH (°)				
	**Skeletal proportional**	**Dental linear**	**Soft tissue proportions**	
	P-A face height (S-Go/N-Me) (%) PFH:AFH (%)	Overjet (mm) Overbite (mm) U1**-**NA (mm)	g'-sn'/sn'-me' (%)	
	S-Ar/Ar-Go (%)	U1 to occlusal plane (mm) U1**-**PP (UADH) (mm)	g'-sn'/sn'-gn' (%)	
	UFH (N-ANS/(N-ANS + ANS-Me)) (%)	U1 to Nasion Perp (mm) L1**-**NB (mm)	Sn-Stomion/Sn-Me (%)	
	LFH/TFH (ANS-Me:N-Me) (%)	L1 protrusion (L1-APg) (mm)		
	Face Ht ratio (N-ANS/ANS-Me) (%) SN/GoMe (%)	L1 to occlusal plane (mm) L1**-**MP (LADH) (mm)		
	ANS-PNS/Me-Go (%)	L1 Tip**-**VRP (mm)		
		U6**-**PT Vertical (mm) U6**-**PP (UPDH) (mm)		
		L6**-**MP (LPDH) (mm)		
		Molar relation (mm)		

The skeletal variables underwent PCA to generate the best suited axis model to find the most significant components that might explain most of the variance found in our data set regarding skeletal craniofacial structures, reducing the dimensionality of the data and ranking them by weighted importance. PCA was conducted to reduce the number of initial variables used in the cephalometric analysis (n = 102) to a smaller set of axes correlated to each other. To assess the components, the axes were rotated so that each axis had large correlations with few variables. The varimax rotation was used and Kaiser normalization was applied to prevent variables with more explanatory capacity from affecting the rotation (IBM SPSS Statistics version 25.0).

## Statistics

### Reliability, accuracy, and method error

The same operator obtained 102 radiographic measurements of each patient. The reliability of the measurements was tested using a replica of the measurements of 15 patients randomly selected and separated in time by a 3-week interval. Specifically, the method error (ME) was calculated once all of the cephalometric traces were completed using the intraclass correlation coefficient, two-way mixed effects model for absolute agreement ICC^15^. The paired Student’s t-test with a *p* value > 0.05 considered non-statistically significant differences between the original and repeated measurements. The accuracy of the method was calculated using the Dahlberg formula in the replicated sample^16^.

### Sub-phenotypes characterization and clusters configuration

**Figure 1:**
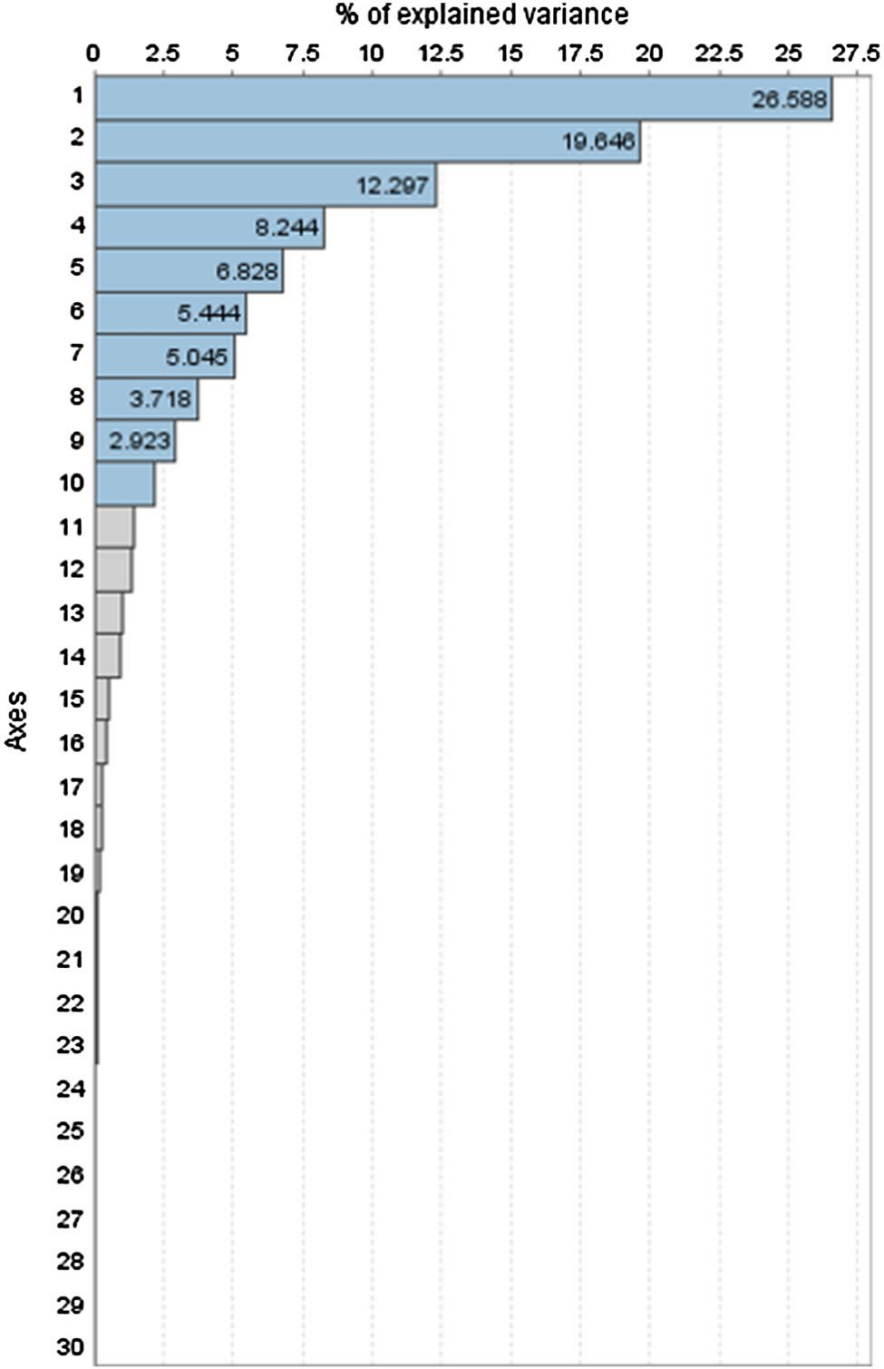
Principal component analysis. Ten principal components accounted for 92.7% of the variation as assessed by Varimax.

**Figure 2:**
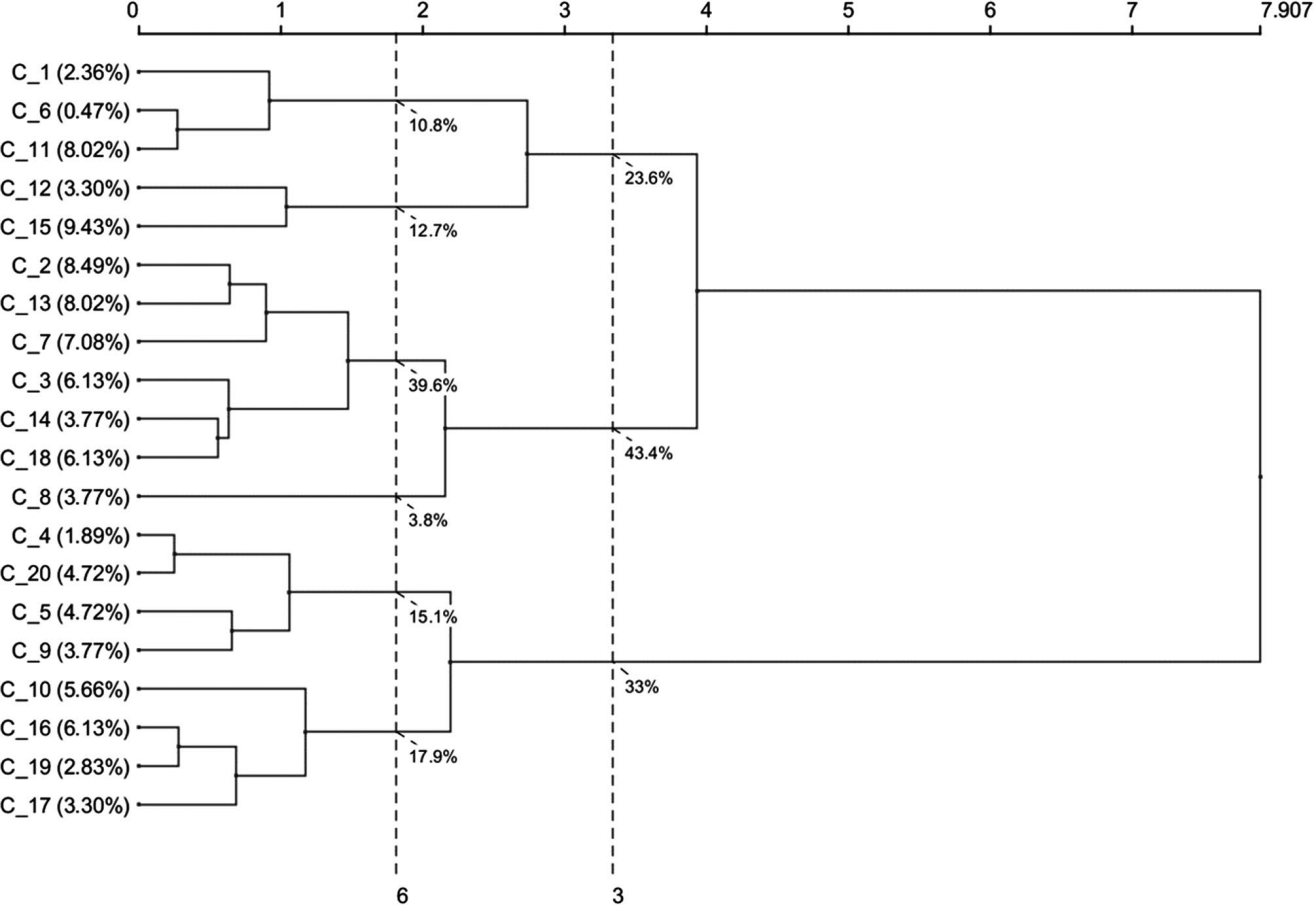
Dendogram using Ward’s method.

A priori data processing explained 92.7% of the variance using 10 main axes (Fig. 1). These enabled the application of cluster analysis and other multivariate techniques. The cluster analysis was performed (Coheris Analytics SPAD version 9.1) to identify and group subjects with similar phenotypes to make comparisons between them. A best suited model of 6 clusters was defined based on Ward’s criterion, defining the distances between groups and establishing the least possible dispersion within groups to ensure the greatest homogeneity for each cluster (Fig. 2). To obtain a graphical representation, the generated skeletal cluster model was represented using the mean value of the main explanatory cephalometric variables of each component.

The potential interaction of CVMS^14^ and sex on each of the generated clusters was also evaluated using the chi-square test. The supplementary cephalometric variables measured on the lateral cephalometric radiographs (dental, soft tissues, and airways) were recorded to observe how those variables produced each cluster.

## Results

### Reliability and accuracy of the method

Reliability tests of the cephalometric measurements determined a *p* value greater than 0.05 and ICC values higher than 0.8, with only one variable less than 0.85 (ANS-PNS/Me-Go (%)), indicating good reliability. The accuracy of the measurements retrieved an error value ranging from 0.02 mm for basal width and 0.2 mm for Wits (FOP) to a maximum value of 6.2 mm for anterior face height (N-Me) and 5.9 mm for length of the mandibular base (Go-Pg). For the angular radiographic measurements, we found error values of 0.02° for the facial taper and 0° for the inferior gonial angle (N-Go-Me) and maximum values of 1.5° for occlusal plane at SN and 1.2° for the articular angle. For the proportional measurements, we found errors of 0 to 3.1% for face height ratio (N-ANS/ANS-Me), ANS-PNS/Me-Go, and S-Ar/Ar-Go, respectively.

### Study sample distribution and types of axis models generated

A total of 699 lateral cephalometric radiographs were initially collected and revised. Of these, 487 had to be rejected due to lack of visible cephalometric points, poor quality, impacted teeth, retained teeth, dental agenesis, and patients with remaining growth or previous orthodontic treatment. Hence, a total of 212 subjects (97 men and 115 women) were included in the present research. Mean age was 28 ± 10.66 years for men and 29 ± 10.12 years for women. All the subjects were CVMS IV or V. Of the total sample, 45 subjects were CVMS IV and the remaining 167 were CVMS V. The total sample showed a mean ANB value of − 1.3 ± 1.5 and a Wits appraisal of − 5.2 ± 3.2. The MP-FH and y-axis variables, two of the most significant in the sample, had averages of 20° ± 5.9° and 66.25° ± 3.9°, respectively.

A 10-axis model was generated from the radiographic skeletal variables via PCA (Fig. 1). The results of PCA revealed that the first 10 principal components explained 92.7% of total variation. Specifically, the first three axes represented almost half of the variation. These axes were represented by sagittal and vertical measurements that reflected cranial, mandibular, maxillary, and facial distances and positions. PC1 represented 26.6% of total variation and was characterized mainly by anteroposterior and vertical linear variables such as N-Me, SN, N-ANS, Co-Gn, and Ar-Gn. PC2 (19.6%) was represented by angular measurements, essentially describing the vertical dimension (N-Go-Me, facial taper, SN-GoGn, and MP-FH). PC3 represented 12.3% of the variance, with measurements indicating the maxillary-mandibular antero-posterior position and vertical dimension (SNA, SNB, and y-axis). PC4 (8.2%) was represented by measurements indicating the antero-posterior position of the maxilla and mandible (ANB and A-NPg). PC5, which explained 6.8% of variation, was typified by the maxillary-mandibular antero-posterior position and vertical dimensions (A-N perp, Pg-N perp, and occ plane to FH). PC6 represented 5.4% of variability and was mostly characterized by proportional measurements indicating the proportions of the anterior facial height. PC7, PC8, PC9, and PC10 represented 13.8% of total variability and each was described by 2 to 3 variables. PC7 (5%) was represented by 3 angular variables (upper gonial angle, articular angle, and Rp-FH). PC8 (3.7%) was represented only by the Wits appraisal and Wits (FOP). PC9 (2.9%) was represented by variables indicating the relationship between mandibular length, anterior cranial base and maxilla (SN/GoMe and ANS-PNS/Me-Go). PC10 represented the lowest percentage of variation with 2.2% of total variance (S-Ar and S-Ar/Ar-Go). A detailed description of the 10 main axes is provided in Table 2.

**Table 2:** Principal components generated based in a ten axes model.

Principal component	PC 1	PC 2	PC 3	PC 4	PC 5	PC 6	PC 7	PC 8	PC 9	PC 10
% of explained variance	26.6	19.6	12.3	8.2	6.8	5.4	5.0	3.7	2.9	2.2
Cumulated % of explained variance	26.6	46.2	58.5	66.8	73.6	79.0	84.1	87.8	90.7	92.9
Cephalometric variables^a^	Anterior cranial base (SN) (mm)	SN**-**GoGn (°)	FH**-**SN (°)	ANB (°)	Occ plane to FH (°)	Cranio-Mx base/SN-palatal plane (°)	Upper Gonial angle (Ar-Go-Na) (°)	Wits (FOP) (mm)	SN/GoMe (%)	Posterior cranial base (S-Ar) (mm)
	Anterior face height (NMe) (mm)	Facial axis-Ricketts (NBa-PtGn) (°)	SNA (°)	N-A-Pg (°)	Facial angle (FH-NPg) (°)	UFH (N-ANS/(N-ANS + ANS-Me)) (%)	Articular Angle (°)	Wits Appraisal (mm)	ANS-PNS/Me-Go (%)	S-Ar/Ar-Go (%)
	Upper face height (N-ANS) (mm)	FMA (MP-FH) (°)	SNB (°)	Convexity (A-NPg) (mm)	Maxillary skeletal (A-N Perp) (mm)	LFH/TFH (ANS-Me:N-Me) (%)	Rp-FH (°)			
	Lower face height (ANS-Me) (mm)	Lower face height (ANS-Xi-Pm)(°)	SND (°)	Pg**-**NB (mm)	Mand. skeletal (Pg-Na Perp) (mm)	Face Ht ratio (N-ANS/ANS-Me) (%)				
	Posterior Face Height (SGo) (mm)	Facial Taper (°)	Y-axis (SGn-SN) (°)							
	Ramus height (Ar-Go) (mm)	Gonial/jaw angle (Ar-Go-Me) (°)	Occ plane to SN (°)							
	Co-Go (mm)	Lower gonial angle (N-Go-Me) (°)	Saddle/sella angle (SN-Ar) (°)							
	Midface length (Co-A) (mm)	Basal width (mm)	Superior angle SN-AB (°)							
	Ar**-**A (mm)	P-A Face height (S-Go/N-Me) (%)								
	Maxillary length (ANS-PNS) (mm)	PFH:AFH (%)								
	Mandibular body length (Go-Gn) (mm)									
	Length of mand base (Go-Pg) (mm)									
	Mandibular length (Co-Gn) (mm)									
	Co-B1 Total mand (mm)									
	Ar**-**Gn (mm)									
	Mx/Md diff (Co-Gn**-**Co-A) (mm)									

^a^variables with the highest contribution in each PC.

### Skeletal class III sub-phenotypes: cluster characterization

**Figure 3:**
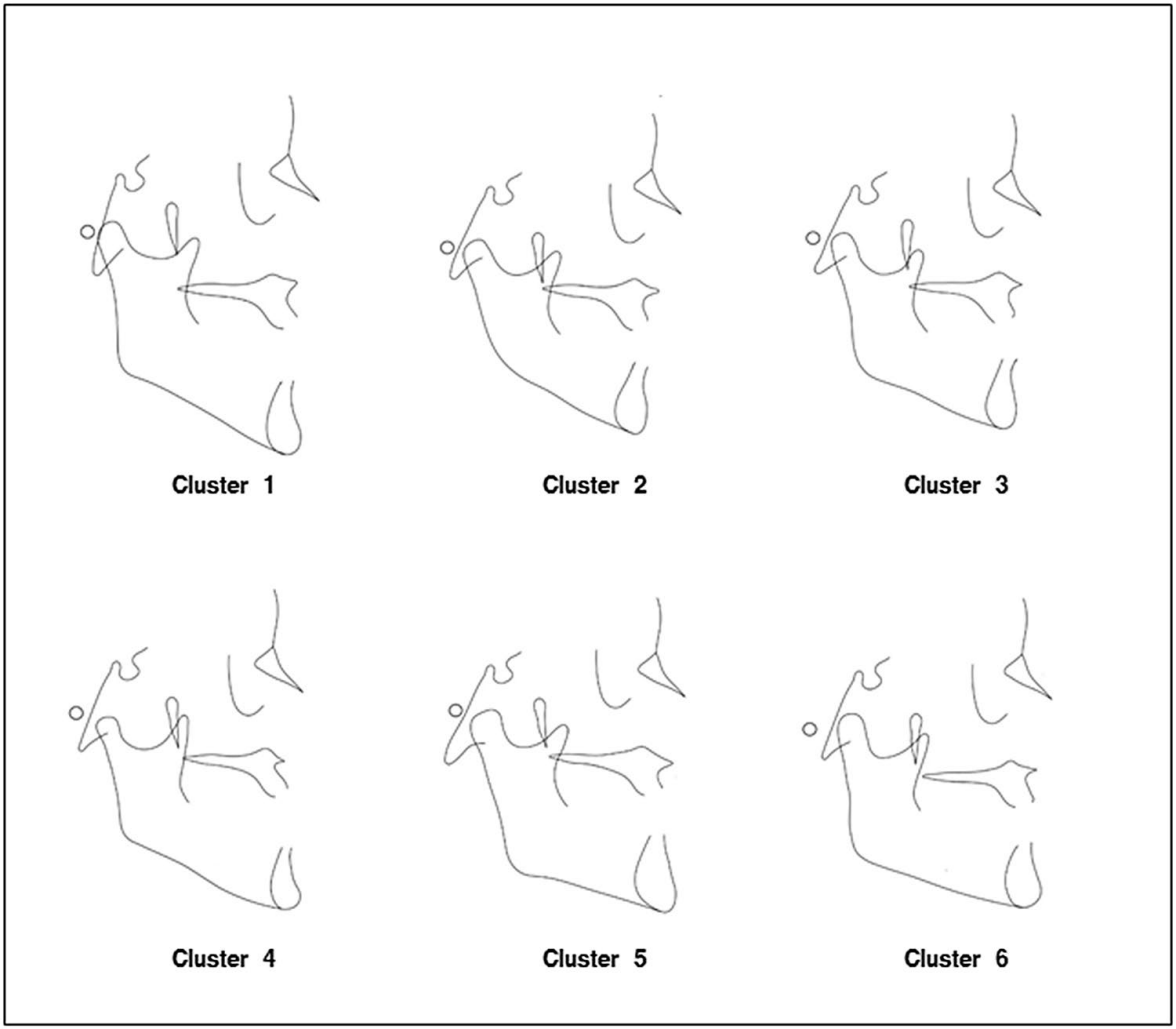
Cluster representation. Cephalometric tracings of the means of each cluster as explained in the text.

The 10-axis model for radiometric variables led to a cluster configuration of a total of six skeletal class III malocclusion subphenotypes, or clearly distinguishable clusters (Fig. 3). The first skeletal cluster (C1) represented 9.9% (n = 21; mean age: 29.9 ± 10.39 years) of the sample. This skeletal class III malocclusion phenotype was characterized by an increased total anterior facial height, enlarged posterior cranial base and larger mandible size than the other groups (Fig. 3).

The second skeletal cluster (C2) represented 18.9% of the sample (n = 40; mean age: 27.25 ± 8.47 years) and showed the vertical component. This group had an increased mandibular plane, as well as a lower proportion of posterior facial height relative to anterior facial height. Bi-maxillary retrusion was also found in this group. Both the upper and lower jaws were positioned in a more posterior location.

The third skeletal cluster (C3) was the most frequent phenotype in the sample, with 33% (n = 70; mean age: 28.49 ± 9.39 years) presenting characteristics intermediate between C2 and C4. C3 was described as a slight skeletal class III malocclusion. In this phenotype, reduced upper facial height was found and the smallest mandibular length of all groups. Midface distance and maxillary length were the second smallest of the six skeletal groups after C4. Slight mandibular projection was also shown. This cluster represented a boundary class III skeletal malocclusion (Fig. 3).

The fourth skeletal cluster (C4) determined in this study represents the least frequent distribution of the skeletal class III cohort, 3.77% (n = 8; mean age: 28.64 ± 9.80 years) and exhibited a severe skeletal class III malocclusion with a reduced anterior and posterior cranial base. The variables for mandibular projection presented the most augmented values of all the skeletal clusters. In addition, upper maxilla hypoplasia was observed. This skeletal C4 had a more decreased posterior facial height than the other clusters and a severely reduced symphysis width (Fig. 3).

The fifth skeletal cluster (C5) (16%, n = 34), unlike C4 was characterized by increased maxilla size and increased length of the middle third of the face compared to the five other skeletal clusters. The mandibular structure had larger dimensions. And the variables characterizing the vertical dimension indicated a brachial phenotype, but of lesser degree than C6 (Fig. 3).

Finally, skeletal cluster 6 (C6) (16%, n = 34) represented a moderate skeletal class III malocclusion with a short face. Skeletal C6 had a considerably reduced anterior facial height (N-Me), a similarly decreased lower facial third (ANS-Me), a smaller mandibular plane (SN-GoGn and MP-FH), a very diminished gonial angle (Ar-Go-Me), and an increased NBa-PtGn angle. Table 3 shows the mean values of the skeletal radiographic variables of each of the groups.

**Table 3:** Craniometric mean values defined in each skeletal cluster.

Cephalometric measurements	Global		C1 (n = 21)		C2 (n = 40)		C3 (n = 70)		C4 (n = 8)		C5 (n = 34)		C6 (n = 39)	
	Mean	SD	Mean	SD	Mean	SD	Mean	SD	Mean	SD	Mean	SD	Mean	SD
**Skeletal angular**														
FH**-**SN (°)	10.77	2.60	10.31	2.84	11.88	2.78	10.69	2.24	12.65	3.13	9.26	2.42	10.95	2.07
SNA (°)	80.14	3.35	79.72	2.43	76.78	3.00	80.04	2.61	80.54	2.95	82.93	2.90	81.49	2.65
SNB (°)	81.49	3.60	80.83	2.72	77.46	2.79	81.12	2.40	85.55	3.37	84.64	2.90	83.09	2.60
ANB (°)	− 1.35	1.52	− 1.09	1.11	− 0.69	0.90	− 1.07	1.06	− 5.01	2.25	− 1.71	1.59	− 1.61	1.48
SND (°)	79.62	3.60	79.44	3.03	75.36	2.57	79.10	2.20	83.94	2.86	82.61	2.74	81.54	2.70
Y-Axis (SGn**-**SN) (°)	66.25	3.97	68.74	2.53	70.81	3.44	66.74	2.15	63.89	2.41	62.75	2.62	62.91	2.60
SN**-**GoGn (°)	28.62	6.42	32.38	3.81	36.36	4.78	29.08	3.24	32.05	3.30	22.83	3.88	22.21	4.11
Cranio-Mx base/SN-palatal plane (°)	8.84	3.40	7.17	3.42	11.47	3.06	8.44	2.48	9.49	3.56	7.08	3.40	9.16	3.39
Occ plane to SN (°)	14.27	4.72	15.61	4.12	19.43	4.34	14.22	2.73	16.71	3.81	10.34	3.92	11.25	3.30
Occ plane to FH (°)	3.50	4.23	5.30	4.45	7.55	4.17	3.53	2.88	4.08	3.28	1.09	3.42	0.30	2.71
Facial axis-Ricketts (NBa-PtGn)(°)	90.78	4.42	86.57	3.10	86.74	3.66	90.08	2.75	93.01	2.08	94.34	2.97	94.87	2.98
FMA (MP-FH) (°)	20.00	5.96	24.25	3.96	26.57	4.74	20.40	3.40	21.65	3.39	15.89	3.46	13.52	4.02
Lower face height (ANS-Xi-Pm) (°)	43.14	4.50	48.27	2.94	46.58	4.15	43.83	2.33	43.48	1.77	40.91	2.98	37.52	2.51
Facial angle (FH-NPg) (°)	93.59	3.29	92.90	2.74	90.08	2.54	92.88	2.19	99.49	1.91	95.39	2.00	96.04	2.39
N-A-Pg (°)	− 5.66	4.14	− 5.80	2.57	− 3.01	2.66	− 4.52	3.07	− 12.93	3.78	− 6.70	4.65	− 7.93	4.12
Facial taper (°)	66.41	4.90	62.88	4.08	63.36	4.68	66.72	3.89	58.88	2.70	68.71	3.22	70.44	3.51
Gonial/jaw angle (Ar-Go-Me) (°)	122.25	7.38	124.17	7.13	128.97	5.96	121.33	5.14	129.84	6.10	120.69	6.13	115.77	6.19
Upper gonial angle (Ar-Go-N) (°)	49.30	4.16	46.31	3.65	50.84	4.50	48.52	3.32	51.69	3.67	50.78	3.97	48.98	4.30
Lower gonial angle (N-Go-Me) (°)	72.95	5.73	77.84	4.36	78.14	5.18	72.82	3.60	78.18	2.95	69.91	3.62	66.80	3.50
Articular angle (°)	143.30	6.37	148.12	5.25	141.79	6.14	144.93	5.82	143.41	7.05	139.46	5.37	142.65	6.19
Saddle/sella angle (SN-Ar) (°)	125.22	5.05	122.26	5.19	127.69	4.71	124.83	4.82	121.06	4.97	125.00	4.53	126.03	4.62
Superior angle SN-AB (°)	83.57	4.94	82.39	3.61	78.53	3.01	82.73	2.89	92.49	5.32	87.31	4.42	85.80	4.10
Rp-FH (°)	77.75	4.82	80.06	5.45	77.60	5.23	79.06	3.95	71.81	2.90	75.21	4.15	77.75	4.30
**Skeletal linear**														
Anterior cranial base (SN) (mm)	68.33	4.83	71.47	4.17	68.80	4.65	65.58	3.39	64.54	4.94	73.19	3.73	67.64	3.83
Anterior face height (NMe) (mm)	119.00	9.56	135.29	7.74	123.17	7.23	114.30	5.14	114.64	3.85	123.30	5.35	111.54	7.70
Upper face height (N-ANS) (mm)	52.50	4.03	57.22	3.36	54.15	3.47	50.08	2.75	50.19	1.85	53.94	3.34	51.84	4.16
Lower face height (ANS-Me) (mm)	67.08	6.79	78.64	5.69	69.89	5.30	64.84	3.75	64.55	3.97	69.80	3.46	60.16	4.54
Posterior cranial base (S-Ar) (mm)	33.88	3.65	37.74	3.64	33.47	3.01	32.36	2.62	28.69	2.98	36.76	3.10	33.49	2.86
Posterior face height (SGo) (mm)	80.63	7.80	89.10	5.86	76.47	5.35	77.02	4.76	72.56	3.82	89.49	6.71	80.76	6.20
Ramus height (Ar-Go) (mm)	51.00	5.96	54.90	4.82	47.42	4.28	48.34	4.13	47.59	2.27	58.39	4.89	51.62	5.00
Co-Go (mm)	66.04	7.08	73.74	4.73	61.73	4.76	62.50	4.48	61.38	2.76	74.85	5.53	65.97	5.22
Convexity (A-NPg) (mm)	− 2.74	2.00	− 3.24	1.45	− 1.55	1.42	− 2.11	1.43	− 6.14	1.76	− 3.39	2.34	− 3.58	1.93
Maxillary skeletal (A-N Perp) (mm)	0.88	3.03	0.14	2.89	− 1.53	2.88	0.72	2.51	3.15	3.56	2.27	2.63	2.35	2.40
Midface length (Co-A) (mm)	84.42	6.23	88.72	5.75	83.20	4.88	80.76	4.12	78.38	4.38	92.39	4.18	84.21	4.58
Ar**-**A (mm)	83.50	6.26	86.40	5.57	82.38	4.90	79.74	3.91	76.63	4.79	91.76	4.56	84.03	4.52
Maxillary length (ANS-PNS) (mm)	51.44	3.82	54.11	4.10	51.13	2.93	49.44	3.05	47.39	3.06	55.06	2.97	51.60	3.03
Pg**-**NB (mm)	2.58	1.97	3.95	1.80	1.48	1.55	2.03	1.86	2.43	1.12	3.02	2.10	3.62	1.59
Mand. skeletal (Pg-N Perp) (mm)	6.85	6.43	6.55	6.66	0.07	5.24	5.41	4.13	17.88	3.81	10.79	4.01	10.86	4.41
Mandibular body length (Go-Gn) (mm)	83.16	6.54	90.06	6.46	80.55	4.82	79.43	3.94	82.56	5.52	88.75	6.15	84.08	5.76
Length of mand base (Go-Pg) (mm)	72.16	5.23	77.17	6.61	70.43	3.74	69.83	3.44	71.16	6.43	75.36	5.09	72.84	4.76
Mandibular length (Co-Gn) (mm)	120.04	8.97	133.03	8.82	117.95	5.35	114.40	5.12	118.88	5.96	129.98	5.32	116.88	6.19
Co-B1 Total mand (mm)	117.73	8.72	130.42	8.66	116.02	5.36	112.38	5.07	117.05	5.90	127.07	4.92	114.23	6.32
Ar**-**Gn (mm)	113.57	8.50	123.75	8.99	111.50	5.27	107.96	4.83	112.84	6.33	123.70	5.38	111.60	5.91
Basal width (mm)	7.30	1.55	7.01	1.34	7.09	1.59	6.85	1.40	5.69	0.72	8.44	1.48	7.82	1.27
Mx/Md diff (Co-Gn**-**Co-A) (mm)	35.62	5.33	44.31	4.67	34.75	5.03	33.63	3.32	40.51	2.79	37.58	3.91	32.67	4.08
Wits (FOP) (mm)	− 6.24	3.48	− 7.21	4.05	− 6.79	4.07	− 5.66	2.48	− 13.34	3.03	− 6.01	2.89	− 4.94	2.53
Wits appraisal (mm)	− 5.25	3.18	− 6.31	3.28	− 5.53	3.61	− 4.49	2.31	− 13.10	3.24	− 5.21	2.29	− 4.18	1.98
**Skeletal proportional**														
P-A face height (S-Go/N-Me) (%)	67.86	5.25	65.90	3.31	62.12	3.37	67.38	2.96	63.33	2.82	72.55	4.10	72.48	4.40
PFH:AFH (%)	55.57	4.79	54.55	2.91	50.16	3.36	54.68	2.95	53.58	2.77	60.67	3.08	59.23	3.76
S-Ar/Ar-Go (%)	66.96	8.08	69.16	8.32	71.05	8.30	67.40	7.80	60.44	6.41	63.18	5.30	65.41	7.82
UFH (N-ANS/(N-ANS + ANS-Me)) (%)	43.97	2.13	42.14	1.70	43.69	1.97	43.59	1.65	43.78	1.97	43.59	1.75	46.28	1.80
LFH/TFH (ANS-Me:N-Me) (%)	56.07	2.14	57.89	1.72	56.11	2.07	56.45	1.69	56.41	1.98	56.56	1.74	53.90	1.84
Face Ht ratio (N-ANS/ANS-Me) (%)	0.79	0.08	0.73	0.06	0.78	0.07	0.77	0.06	0.78	0.07	0.77	0.06	0.87	0.07
SN/GoMe (%)	100.50	7.31	98.14	8.17	103.36	8.29	99.52	6.54	95.14	8.36	103.42	5.14	99.14	6.48
ANS-PNS/Me-Go (%)	0.76	0.06	0.74	0.07	0.78	0.07	0.75	0.06	0.69	0.06	0.77	0.05	0.75	0.06

*SD* standard deviation.

With respect to the distribution of gender and skeletal maturation stage in the generated clusters, the CVMS^14^ was homogenous for all clusters (*p* > 0.05), while gender was asymmetrically distributed in four of the six clusters. C1, C3, C4, and C5 had differences in gender distribution (*p* < 0.05). C1 and C5 were mostly represented by male subjects, at 85.7% and 79.4%, respectively, while C3 and C4 were represented mainly by female subjects, at 74.3% and 100%, respectively. Similarly, in the male-dominated clusters, total linear measurements (Go-Gn, Go-Pg, NMe, SN, S-Ar, and ANS-PNS) were much higher than in the female-dominated clusters (C3 and C4).

With respect to the supplementary soft tissue variables measured, the position of Pg' with respect to the vertical N' followed the underlying bone structure, with the position of Pg' coinciding with the position of bone Pg in all clusters. The greatest distance from this point to the vertical N in the tissues was observed in C4 hard tissues (17.87 ± 3.80), as well as the soft tissues (22.55 ± 4.30). The group with the second longest distance from Pg to the perpendicular N in both hard tissues (10.85 ± 4.41) and soft tissues (16.87 ± 3.88) was C6. The same relationship was observed in an analogous way in the rest of the groups.

Similarly, facial height in the soft tissues (Sn'–Me') corresponded to facial bone height (ANS-Me), except in two sub-groups, C2 and C5, where C5 had the second largest lower facial height in the soft tissues (72.96 ± 3.78) and C2 the third (72.23 ± 5.06), while the opposite was the case in the skeletal tissue, where C2 (69.89 ± 5.30) had the second lowest increased facial height and C5 the third (69.80 ± 3.45) with a difference of 0.09 mm.

The position of the upper lip with respect to the soft vertical through the N' point coincided with the position of the upper incisors with respect to the perpendicular through N in the 6 subphenotypes, with the largest distance being shown in C6 and the least in C2. The most retruded lower lip was presented in C6, both with respect to the S line and the -E line. The least retracted position of the lower lip with respect to the S line was presented by C4 and C2 with respect to the -E line.

The analysis of the dental component indicated that only one of the sub-phenotypes (C4) had an anterior crossbite (− 2.6 ± 2.75). The rest had a positive overjet, which was greatest in C6 (1.8 ± 2.24). This subgroup also had the highest overbite (2.45 ± 1.93), while C1 had the lowest (0.34 ± 1.77) of the 6 groups. C4 had the highest retroinclination of the lower incisor (L1-FH: 82.55 ± 10.32 and L1-MP: 75.8 ± 9.48), while the highest vestibular inclination of the upper incisor was found in C5 (U1-SN: 109.66 ± 4.88 and U1-palatal plane: 116.75 ± 5.05) and the smallest in C2 (U1-SN: 100.43 ± 8.04, U1-palatal plane: 111.89 ± 7.26, and U1-NA: 23.65 ± 6.82). In the same context, in C6, both the upper and lower incisors were less protruded (U1-NA: 4.92 ± 1.82, U1 to N perp: 8.09 ± 3.61, L1-NB: 1.11 ± 2.3, and L1-APg: 0.85 ± 2.63), while in C1 (U1-NA: 7.14 ± 1.98 and L1-NB: 4.33 ± 2.49) and C4 (U1 to N perp: 10.68 ± 3.85 and L1-APg: 4.25 ± 2.99), both incisors were more protruded compared to the rest of the sub-phenotypes.

The height of the anterior and posterior nasal cavity increased the most (55.97 ± 4.01 and 89.68 ± 4.44, respectively) in C1 and decreased the most in C4 (45.31 ± 1.94 and 76.04 ± 1.78, respectively), while the upper and lower airways decreased in C3 (9.81 ± 3.16 and 13.6 ± 2.78, respectively) and the largest dimension was found in C5 (12.68 ± 6.02 and 16.49 ± 6.80, respectively). The remaining means of the supplementary variables are shown in Supplementary Table 3.

## Discussion

In the present study, 6 different types of class III skeletal malocclusion phenotypes and 10 main components were obtained using grouping techniques. Both approaches, principal component generation and morphological class III cluster generation, form part of a sequential process in which the principal component axes are not real biological coordinates of the system but a means of grouping the variables for later construction of the class III clusters. Specifically, the analysis of the main components facilitated the generation of 10 axes, which were constructed exclusively from the 55 skeletal measurements of the subjects of the study. This allowed for extremely high coverage of variance (92.7% of the variance). We found differences in the size of the axis models generated compared with previously published studies^5,8,17^, which restricted the number of main components to five^17^ and six^5,8^, respectively, and covered 67%^17^ to 81.2%^5^ of total variance in their sample. This may be because in the present study, only skeletal measurements were considered for the axis model generation.

Dental and soft tissue measurements were excluded to avoid potential interactions with the construction of the purely skeletal class III clusters, as these parameters could modify or mask the underlying craniofacial structure^12^. Hence, using a large number of critical measurements, 55 skeletal variables, we achieved a notable percentage of explained variation, which, although not excellent, shows minimal loss of information, which might be of critical importance for the configuration of skeletal clusters, although assuming, as with other diagnostic methods, that some uncovered features would be underrepresented.

As described in previously published studies^5,8,17^, the first three axes represented more than half [58.5%] of the total variation found in the sample, essentially referring to the description of sagittal and vertical parameters, while others^5,8,17^ also represented the position of the lower incisor in the first half of the variation derived from the involvement of dental variables in the formation of PCs. Most of the parameters that represent sagittal and vertical measurements in our PC1 (26.6% of variation), PC2 (19.6% of variation), and PC3 (12.3% of variation) are equivalent to the parameters of PC1 (23.7% and 20.6%) and PC2 (17.3% and 19.34%) in other studies^5,8^, while PC1 in another study^17^ is more like PC4 (9.3%) and PC5 (6.5%) (ANB, facial angle, and Pg-N perp) in the present research, despite the fact that the same sagittal and vertical parameters were included in these aforementioned components. The observed differences might be at least partially explained by the type of class III patients included and their distribution, essentially based on distinct ethnic origin and growing stage of the included subjects.

The 10 main models enabled the subsequent generation of skeletal class III malocclusion sub-phenotypes or clusters. According to the dendrogram analysis, potential construction with three or six skeletal clusters was possible (Fig. 2). Nevertheless, a six-cluster skeletal model was selected, since we observed substantial loss of representation of clearly detailed sub-phenotypes in the three-cluster model, such as C2 representing a vertical cluster with bimaxillary retrusion, or C4 representing maxillary hypoplasia with a prognathic mandible. The number of clusters found in previous studies that also used cluster analysis focusing on class III malocclusion classification varied between 4^8^ and 7 clusters^2^, very close to the number of clusters established in the present research. Despite this, we also observed in the literature other studies with even more clusters than those found in our study, varying between 3^3,6,7^ and 14 clusters^4^, which might provide high variation in coverage but completely lose efficiency in terms of practical utility in clinical settings. Other authors^3,4,6,7^ used a diffuse cluster analysis when establishing a three-cluster model^6,7^, implying that the elements of analysis may belong to more than one cluster, while others used a hierarchical cluster analysis^3^ instead of the mixed cluster analysis used in the present research.

However, with respect to 14 different clusters^4^, the increased number of clusters could be derived, at least in part, from the inclusion of class III dental malocclusions as well as skeletal class III malocclusions, and the classification was also used to evaluate the effects of treatment^4^, which required large numbers in order to provide enough patients in each sub-group, while at the same time ensuring potentially higher homogeneity in intersubject characteristics in each of the constructed groups.

With respect to the skeletal clusters generated in the present research, the cluster representing the highest percentage of the sample was C3, at 33%. This cluster was characterized by a mesofacial pattern (y-axis: 66.7 ± 2.1, SN-GoGn: 29.0 ± 3.2, and NBa-PtGn: 90.1 ± 2.7) with slight maxillary retrusion (convexity: − 2.1 ± 1.4) and a slight anterior mandibular position (PgN perp: 5.4 ± 4.1) but with decreased total mandibular size (GoGn: 79.4 ± 3.9, GoPg: 69.8 ± 3.4, and CoGn: 114.4 ± 5.1) than the rest of the clusters. The characteristics of this cluster were similar to those found in C2 clusters in previous studies^5^, which was one of the most representative clusters in their sample. In the same research^5^ and a previous study^17^, the group with the highest number of subjects was represented by a group characterized by a retrusive jaw (C5 and C3, respectively). In our study, the retrusive jaw model was the second most representative model in the sample (C2), representing 18.8% of the total sample. In another study, on the other hand, the model with maxillary deficiency alone, but without mandibular compromise, was not represented^8^. Interestingly, this must have derived from the ethnic origin of the sample population, since in studies that analyzed Caucasian subjects^5^ and even a mixed sample with the highest percentage of subjects being of Caucasian origin^17^, this type of cluster was represented, as in the present research. In contrast, clusters generated from samples of Asiatic origin did not generate this type of sub-phenotype^8^, which clearly demonstrates the morphological differences between ethnic groups^11,18,19^ and even between different populations of the same ethnic group when it comes to analyzing class III malocclusion sub-phenotypes.

In our study, the soft tissue, dental, and airway variables were excluded to avoid an inadequate interpretation of the generated groups, since it has been observed that soft tissues do not closely follow bone structures^12^. Despite this, several common characteristics related to the skeletal parts and soft tissues were observed.

In this respect, the soft tissue of the chin continued the underlying bone structure in the 6 sub-phenotypes found. Similarly, lower facial height in soft tissues (Sn'-Me') and hard tissues (ANS-Me) coincided in 4 (C1, C3, C4, and C6) of the 6 clusters. Conversely, skeletal facial height in C2 (69.89 ± 5.30) was 0.09 mm higher than in C5 (69.80 ± 3.45), but facial height in soft tissues (72.23 ± 5.06) was 0.73 mm lower than in C5 (72.96 ± 3.78). Despite this slight difference, our results agree with previous observations^12^, which concluded that the soft tissue of the lower facial third continues the skeletal profile, but not in the labial region^12^. In this study, as observed in previous ones^20–22^, a more significant relationship was found between the labial tissue and the position of the incisors.

The position of the lower lip with respect to the lower incisor was analyzed in several studies that found a close relationship between the two^23,24^, even observing a more significant relationship in the position of the lower incisor with respect to the lower lip compared to the relationship between the upper incisor and the upper lip^23^. This study found that in 5 of the 6 sub-groups, the position of the lower lip coincided with the position of the lower incisor. In the case of C6, the most retruded position of the lower incisor was observed with both the NB line (1.1 ± 2.3) and A-Pg (0.84 ± 2.62), coinciding with the most retruded position of the lower lip with respect to the S line (− 2.62 ± 2.22) and E line (− 5.29 ± 2.43). The relationship between the position of the lower incisor with the NB plane or A-Pg plane with respect to the position of the lower lip with the S line and/or E line was observed in C5, C4, C3, and C2, but not in C1. Individual characteristics may lead to different soft tissue behaviors with respect to the bones and/or teeth^23–25^, with different correlations between the two components. The method used for the analysis of soft tissues should also be taken into account, since recent studies support the need to conduct this analysis using facial photographs, since skeletal cephalometry and soft tissue cephalometry lead to different diagnoses of the patient’s facial characteristics^23^.

Due to the considerable variations, caution must be taken when analyzing the soft profile, especially at the labial level. Parameters such as labial thickness or nasal size, which can vary widely in each individual^25^ and do not follow a skeletal pattern^26^, cannot be ignored. These variations, not only individual, but also soft tissue variations between genders^25^, can lead to a distortion of the final labial position.

In previously published articles^3,5–8,17^ that conducted this type of classification of skeletal class III malocclusion, the samples contained similar gender percentages to our own sample: 40.8% were males and 58.6% were females. In other studies, the gender mix in the total sample ranged from 40.2%^8^ to 48.7%^3^ for males, and between 51.3%^3^ and 59.7%^8^ for females. Only one of the aforementioned studies^8^ mentioned the gender percentage in each of the clusters found: two of the groups found in that study (C3 and C4) had substantially higher distributions of women than men. In our study, we observed another 3 clusters in which the difference in the female/male ratio was greater than 40% (C1: 14.3/85.7, C3: 74.3/25.7, and C5: 20.6/79.4). Although both clusters followed a similar trend in terms of gender distribution, a thorough analysis of the cluster characteristics found some differences in the configuration of skeletal structures derived from ethnicity of Asiatic and Caucasian origin^18,27,28^.

Beyond differences in ethnicity and gender, skeletal class III malocclusion is strongly linked to genetic components and environmental factors^29^. Current phenotype and genotype studies of this malocclusion^30–33^ have been reported in the literature to develop preventive and therapeutic advances in these patients^34,35^. Recent studies in the field of medicine focus on the search for preventive measures and individualized treatments based on the patient’s morphological and genetic components^34,36–38^. However, this will require future research, larger samples, and collaborative multicenter studies^5^ to better understand the differences between regions and environmental factors involved. To reach this point, and bearing in mind that the distribution and frequencies of some specific genetic variations can differ substantially between different populations^39^, it is imperative to obtain an accurate and precise classification in the sub-phenotype diagnosis of each population and/or ethnic group. The present research offers significant data on skeletal class III malocclusion sub-phenotyping in subjects of southern European origin. To the best of our knowledge, this is the first scientific evidence in the field of skeletal clusters of subjects of European origin affected by class III malocclusion.

## Conclusions

In the present study, 10 axes were responsible for 92.7% of variability derived from 55 pure skeletal radiographic measurements. Similar to the results of other articles, different clusters represented phenotypic variability in class III skeletal malocclusion. The present research identified six clusters that clearly represented different sub-phenotypes of skeletal class III malocclusion in a population of southern European origin.

## Supplementary Tables.

Supplementary Information

## References

[1] Proffit, W.R., White, P.R. & Sarver DM. *Contemporary Treatment of Dentofacial Deformity* (ed. Mosby Elsevier) (St. Louis, 2003).

[2] Hong SX, Yi CK (2001). A classification and characterization of skeletal class III on etio-pathogenic basis. Int. J. Oral Maxillofac. Surg..

[3] Abu Alhaija ES, Richardson A (2003). Growth prediction in Class III patients using cluster and discriminant function analysis. Eur. J. Orthod..

[4] Li S, Xu TM, Lin JX (2009). Analysis of treatment templates of angle’s class III malocclusion patients. West China J. Stomatol..

[5] MorenoUribe LM, Vela KC, Kummet C, Dawson DV, Southard TE (2013). Phenotypic diversity in white adults with moderate to severe Class III malocclusion. Am. J. Orthod. Dentofac. Orthop..

[6] Auconi P, Scazzocchio M, Defraia E, McNamara JA, Franchi L (2014). Forecasting craniofacial growth in individuals with class III malocclusion by computational modelling. Eur. J. Orthod..

[7] Auconi P, Scazzocchio M, Cozza P, McNamara JA, Franchi L (2015). Prediction of Class III treatment outcomes through orthodontic data mining. Eur. J. Orthod..

[8] Li C, Cai Y, Chen S, Chen F (2016). Classification and characterization of class III malocclusion in chinese individuals. Head Face. Med..

[9] Bro R, Smilde AK (2014). Principal component analysis. Anal. Methods.

[10] Everitt BS, Landau S, Leese M, Stahl D (2011). Cluster Analysis.

[11] De Frutos-Valle L, Martin C, Alarcon JA, Palma-Fernandez JC, Iglesias-Linares A (2019). Sub-clustering in skeletal class III phenotypes of different ethnic origins: a systematic review. J. Evid. Based Dent. Pract..

[12] Malá PZ, Krajíček V, Velemínská J (2018). How tight is the relationship between the skeletal and soft-tissue facial profile: a geometric morphometric analysis of the facial outline. Forensic Sci. Int..

[13] World Medical Association (2013). Declaration of Helsinki: ethical principles for medical research involving human subjects. JAMA.

[14] Baccetti T, Franchi L, McNamara JA (2002). An improved versión of the cervical vertebral maturation (CVM) method for the assessment of mandibular growth. Angle Orthod..

[15] Shrout PE, Fleiss JL (1979). Intraclass correlations: uses in assessing rater reliability. Psychol. Bull..

[16] Souza-Galvão MC, Sato JR, Edvaldo Capobiango Coelho EC (2012). Dahlberg formula: a novel approach for its evaluation. Dental Press J. Orthod..

[17] Bui C, King T, Proffit W, Frazier-Bowers S (2006). Phenotypic characterization of class III patients. Angle Orthod..

[18] Barbosa M, Vieira EP, Quintao CC, Normando D (2016). Facial biometry of Amazon indigenous people of the Xingu River – Perspectives on genetic and environmental contributions to variation in human facial morphology. Orthod. Craniofac. Res..

[19] Oh E, Ahn SJ, Sonnesen L (2018). Ethnic differences in craniofacial and upper spine morphology in children with skeletal Class II malocclusion. Angle. Orthod..

[20] Marşan G, Oztaş E, Kuvat SV, Cura N, Emekli U (2009). Changes in soft tissue profile after mandibular setback surgery in Class III subjects. Int. J. Oral Maxillofac. Surg..

[21] Contini E, Orthod D, Campi S, Caprioglio A (2015). Profile changes following lower incisor repositioning: a comparison between patients with different growth pattern. Minerva Stomatol..

[22] Shamlan MA, Aldrees AM (2015). Hard and soft tissue correlations in facial profiles: a canonical correlation study. Minerva Stomatol..

[23] Nucera R (2017). Diagnostic concordance between skeletal cephalometrics, radiograph-based soft-tissue cephalometrics, and photograph-based soft-tissue cephalometrics. Eur. J. Orthod..

[24] Hersberger-Zurfluh MA (2018). Facial soft tissue growth in identical twins”. Am. J. Orthod. Dentofacial Orthop..

[25] Halazonetis, D,J. Morphometric evaluation of soft-tissue profile shape. *Am. J. Orthod. Dentofacial Orthop.* **131,** 481–489 (2007). 10.1016/j.ajodo.2005.06.031 17418714

[26] Holton NE, Alsamawi A, Yokley TR, Froehle AW (2016). The ontogeny of nasal shape: An analysis of sexual dimorphism in a longitudinal sample. Am. J. Phys. Anthropol..

[27] Choi SH (2016). Changes in the craniofacial complex and alveolar bone height of young adults: Applications to dental medicine. Clin. Anat..

[28] Tutkuviene J (2016). Age- and sexrelated growth patterns of the craniofacial complex in European children aged 3–6 years. Ann. Hum. Biol..

[29] Xue F, Wong RW, Rabie AB (2010). Genes, genetics, and Class III malocclusion. Orthod. Craniofac. Res..

[30] Perillo L (2015). Genetic association of ARHGAP21 gene variant with mandibular Prognathism. J. Dent. Res..

[31] Saito F, Kajii TS, Oka A, Ikuno K, Iida J (2017). Genome-wide association study for mandibular prognathism using microsatellite and pooled DNA method. Am. J. Orthod. Dentofac. Orthop..

[32] Cruz CV (2017). Genetic polymorphisms underlying the skeletal Class III phenotype. Am. J. Orthod. Dentofac. Orthop..

[33] Xiong X, Li S, Cai Y, Chen F (2017). Targeted sequencing in FGF/FGFR genes and association analysis of variants for mandibular prognathism. Medicine.

[34] Barelli E (2019). Exploiting the interplay between cross-sectional and longitudinal data in Class III malocclusion patients. Sci. Rep..

[35] Souki, B.Q. et al . Development and validation of a prediction model for long-term unsuccess of early treatment of Class III malocclusion. *Eur. J. Orthod.* cjz031; 10.1093/ejo/cjz031 (2019). 10.1093/ejo/cjz031 31067294

[36] Chamberlain JR, Chamberlain JS (2017). Progress toward gene therapy for duchenne muscular dystrophy. Mol. Ther..

[37] Kucuksezer UC, Ozdemir C, Akdis M, Akdis CA (2018). Precision/personalized medicine in allergic diseases and asthma. Arch. Immunol. Ther. Exp..

[38] Wykes RC, Lignani G (2018). Gene therapy and editing: Novel potential treatments for neuronal channelopathies. Neuropharmacology.

[39] Cunha A (2019). Genetic variants in *ACTN3* and *MYO1H* are associated with sagittal and vertical craniofacial skeletal patterns. Arch. Oral Biol..

