# Supplementary material for: Sub-clustering in skeletal class III malocclusion phenotypes via principal component analysis in a southern European population
Source: Sci Rep. 2020 Oct 21;10:17882. doi: 10.1038/s41598-020-74488-w (PMC7578100; doi:10.1038/s41598-020-74488-w)
Supplement: Supplementary file 1 — Supplementary Information [file 41598_2020_74488_MOESM1_ESM.pdf]

Sub-Clustering in Skeletal Class III Malocclusion Phenotypes via Principal Component Analysis in a Southern European Population

L de Frutos-Valle

C Martín

JA Alarcón

JC Palma-Fernández

R Ortega

A Iglesias-Linares

Supplementary Table 1. **Eligibility criteria.**

**Inclusion criteria**	**Exclusion criteria**
CVMS IV-V (*)	Absence of lateral radiography
Absence of syndromic conditions or dentofacial trauma	Incomplete or low quality lateral radiography
Absence of a history of severe facial	Lack of interest of the study subject
No orthodontic treatment	Patients who did not sign informed consent
No dental agenesis, impacted teeth (with the exception of third molars) or teeth retained.	
And at least 2 of the following inclusion criteria:	
Class III molar or canine without loss of anterior space	
Witts appraisal < -0.5	
ANB ≤ 0º.	

Cervical vertebral maturation stage *(*) (Baccetti et al., 2002)*

Supplementary Table 2. Cephalometric measurements and landmarks.

**SKELETAL ANGULAR**	FH - SN (º)	Angle formed by Frankfort plane (FH) and Sella (S) - Nasion (N) plane
	SNA (º)	Angle formed by Sella (S), Nasion (N) and point A (A)
	SNB (º)	Angle formed by Sella (S), Nasion (N) and point B (B)
	ANB (º)	Angle formed by Nasion (N), point A (A) and point B (B)
	SND (º)	Angle formed by Sella (S), Nasion (N) and point D (D)
	Y-Axis (SGn-SN) (º)	Angle formed by Sella (S) - Gnation (Gn) plane and Sella (S) - Nasion (N) plane
	SN - GoGn (º)	Angle formed by Sella (S) - Nasion (N) plane and Gonion (Go) - Gnation (Gn) plane
	Cranio-Mx Base/SN-Palatal Plane (º)	Angle formed by Sella (S) - Nasion (N) plane and palatal plane
	Occ Plane to SN (º)	Angle formed by occlusal plane and Sella (S) - Nasion (N) plane
	Occ Plane to FH (º)	Angle formed by occlusal plane and Frankfort plane (FH)
	Facial Axis-Ricketts (NBa-PtGn)(º)	Angle formed by Nasion (N) - Basion (Ba) plane and Pterigoid (Pt) - Gnation (Gn) plane
	FMA (MP-FH) (º)	Angle of mandibular plane. Angle formed by mandibular plane (MP) and Frankfort plane (FH)
	Lower Face Height (ANS-Xi-Pm)(º)	Angle formed by the anterior nasal spine (ANS), Xi point and suprapogonion (Pm)
	Facial Angle (FH-NPg) (º)	Angle formed by Frankfort plane (FH) and Nasion (N) - Pogonion (Pg) plane
	N-A-Pg (º)	Angle formed by Nasion (N), point A (A) and Pogonion (Pg)
	Facial Taper (º)	Angle formed by the mandibular plane and facial plane
	Gonial/Jaw Angle (Ar-Go-Me) (º)	Angle formed by the Articulare (Ar), Gonion (Go) and Menton (Me)
	Upper Gonial Angle (Ar-Go-Na) (º)	Angle formed by the Articulare (Ar), Gonion (Go) and Nasion (N)
	Lower Gonial Angle (Na-Go-Me) (º)	Angle formed by the Nasion (N), Gonion (Go) and Menton (Me)
	Articular Angle (º)	Angle formed by Sella (S), Articulare (Ar) and Gonion (Go)
	Saddle/Sella Angle (SN-Ar) (º)	Angle formed by the Sella(S), Nasion (Na) and Articulare (Ar)
	Superior Angle SN-AB (º)	Angle formed by Sella (S) - Nasion (N) plane and point A (A) - point B (B) plane
	Rp-FH (º)	Angle formed by ramus plane (Rp) and Frankfort plane (FH)
**SKELETAL LINEAR**	Anterior Cranial Base (SN) (mm)	Distance between Sella (S) and Nasion (N)
	Anterior Face Height (NMe) (mm)	Distance between Nasion (N) and Menton (Me)
	Upper Face Height (N-ANS) (mm)	Distance between Nasion (N) and Anterior Nasal Spine (ANS)
	Lower Face Height (ANS-Me) (mm)	Distance between Anterior Nasal Spine (ANS) and Menton (Me)
	Posterior Cranial Base (S-Ar) (mm)	Distance between Sella (S) and Articulare (Ar)
	Posterior Face Height (SGo) (mm)	Distance between Sella (S) and Gonion (Go)
	Ramus Height (Ar-Go) (mm)	Distance between Articular (Ar) and Gonion (Go)
	Co-Go (mm)	Distance between Condilion (Co) and Gonion (Go)
	Convexity (A-NPg) (mm)	Distance from point A (A) to Nasion (N)-Pogonion (Pg) line
	Maxillary Skeletal (A-N Perp) (mm)	Distance from point A (A) to the line perpendicular to Frankfort plane via Nasion (N perp)
	Midface Length (Co-A) (mm)	Distance between Condilion (Co) and point A (A)
	Ar - A (mm)	Distance between Articulare (Ar) and point A (A)
	Maxillary length (ANS-PNS) (mm)	Distance between Anterior Nasal Spine (ANS) and Posterior Nasal Spine (PNS)
	Pg - NB (mm)	Distance between Pogonion (Pg) to Nasion (N) - point B (B) line
	Mand. Skeletal (Pg-N Perp) (mm)	Distance between point B (B) to the line perpendicular to the Frankfort plane via Nasion (N perp)
	Mandibular Body Length (Go-Gn)(mm)	Distance between Gonion (Go) and Gnation (Gn)
	Length of Mand Base (Go-Pg)(mm)	Distance between Gonion (Go) and Pogonion (Pg)
	Mandibular length (Co-Gn)(mm)	Distance between the Condilion (Co) and Gnation (Gn)
	Co-B1 Total mand (mm)	Distance between Condilion (Co) and point B (B)
	Ar - Gn (mm)	Distance between Articular point (Ar) and Gnation (Gn)
	Basal Width (mm)	Horizontal distance of the symphysis at point B (B) level
	Mx/Md diff (Co-Gn - Co-A)(mm)	Difference between the Condilion (Co) - Gnation (Gn) distance and the Condilion (Co) - point A (A) distance
	Wits (FOP) (mm)	Distance between point A and point B projected in the functional occlusal plane colusal plane
	Wits Appraisal (mm)	Distance between point A and point B projected in the occlusal plane
**SKELETAL PROPORTIONAL**	P-A Face Height (S-Go/N-Me) (%)	Proportion existing between Sella (S) - Gonion (Go) distance and the Nasion (N) and Menton (Me) distance
	PFH:AFH (%)	Ratio of Posterior Facial Height (PFH) to Anterior Facial Height (AFH)
	S-Ar/Ar-Go (%)	Ratio of posterior cranial base (S-Ar) to ramus height (Ar-Go)
	UFH (N-ANS/(N-ANS+ANS-Me)) (%)	Proportion of upper facial height
	LFH/TFH (ANS-Me:N-Me) (%)	Ratio of Lower Facial Height (LFH) to Total Facial Height (TFH)
	Face Ht Ratio (N-ANS/ANS-Me) (%)	Total Facial Height Ratio
	SN/GoMe (%)	Proportion between Sella (S) - Nasion (N) line and Gonion (Go) - Menton (Me) line.
	ANS-PNS/Me-Go (%)	Ratio of maxillary length (ANS-PNS) to mandibular length (Me-Go)
**DENTAL ANGULAR**	Interincisal Angle (U1-L1) (º)	Angle between upper incisor axisaxis (U1) and lower incisor axis(L1).
	U1 - NA (º)	Angle between upper incisor axis (U1) and Nasion (N)-point A (A) line
	U1 - SN (º)	Angle between upper incisor axis (U1) and Sella (S)-Nasion point(N) line
	U1 - Palatal Plane (º)	Angle between upper incisor axis (U1) and the palatal plane
	U1 - FH (º)	Angle between the upper incisor axis (U1) and Frankfort plane (FH)
	L1 - NB (º)	Angle between lower incisor axis (L1) and Nasion (N)-point B (B) line
	L1 to A-Pg (º)	Angle between lower incisor axis (L1) and point A (A)- Pogonion (Pg) line
	L1 - FH (º)	Angle between lower incisor axis (L1) and Frankfort plane (FH)
	IMPA (L1-MP) (º)	Angle between lower incisor axis (L1) and mandibular plane.
	L6 long axis - MP (º)	Angle between first lower molar axis (L6) and mandibular plane
**DENTAL LINEAR**	Overjet (mm)	Horizontal distance between the incisal edge of the upper incisor (U1) and the lower incisor (L1)
	Overbite (mm)	Vertical distance between the incisal edge of the upper incisor (U1) and the lower incisor (L1)
	U1 - NA (mm)	Distance between upper incisor (U1) and Nasion (N)-point A (A) line
	U1 to Occlusal Plane (mm)	Distance between upper incisor (U1) and occlusal plane
	U1 - PP (UADH) (mm)	Distance between upper incisor (U1) and palatal plane
	U1 to Nasion Perp (mm)	Distance between upper incisor (U1) and the line perpendicular to Frankfort via the Nasion (N)
	L1 - NB (mm)	Distance between lower incisor (L1) and Nasion (N) - point B (B) line
	L1 Protrusion (L1-APg) (mm)	Distance between lower incisor (L1) and point A (A) - Pogonion (Pg) line
	L1 to Occlusal Plane (mm)	Distance between lower incisor (L1) and occlusal plane
	L1 - MP (LADH) (mm)	Distance between lower incisor (L1) and mandibular plane
	L1 Tip - VRP (mm)	Distance between upper incisor (U1) and perpendicular line to the vertical reference plane (VRP: plane built subtracting 7º from the Sella (S)-Nasion (N) line passing through the Sella (S))
	U6 - PT Vertical (mm)	Distance between first upper molar (U6) and vertical Pterigoyd line (vertical PT)
	U6 - PP (UPDH) (mm)	Upper posterior dentoalveolar heigh. Distance from upper first molar (U6) to palatine plane
	L6 - MP (LPDH) (mm)	Lower posterior dentoalveolar heigh. Distance between first lower molar (L6) and palatal plane
	Molar Relation (mm)	
**SOFT TISSUE ANGULAR**	NLA (Nasal Angle) (º)	Angle formed angle between columella (Cm), subnasale (Sn) and labrale superius (Ls)
	Facial Convexity (G'-Sn-Pg') (º)	Angle formed between Glabella (G'), Subnasale (Sn) and soft Pogonion (Pg')
	H-Angle (Pg'UL-Pg'N') (º)	Angle formed between Soft Pogonion (Pg') - Upper lip (UL) line and Soft Pogonion (Pg') - Soft Nasion (N') line
**SOFT TISSUE LINEAR**	Upper Lip - S Line (mm)	Distance between upper lip and S line
	Upper Lip - VRP (mm)	Distance between the upper lip and the perpendicular line to the vertical reference plane (VRP: plane built subtracting 7º from the Sella (S)-Nasion (N) line passing through the Sella (S))
	STissue N Vert (N Perp) to Upper Lip (mm)	Distance between the upper lip and the perpendicular line to the Frankfort plane via the Nasion pointsoft (N')
	Lower Lip - S Line (mm)	Distance between lower lip and S line (esthetic plane of Steiner)
	Lower Lip to E-Plane (mm)	Distance between lower lip and E line (esthetic plane of Ricketts)
	Lower Lip - VRP (mm)	Distance between the lower lip and the perpendicular line to vertical reference plane (VRP: plane built subtracting 7º from the Sella (S)-Nasion (N) line passing through the Sella (S))
	STissue N Vert (N Perp) to Lower Lip (mm)	Distance between lower lip and perpendicular line to Frankfort plane via the soft Nasion (N')
	STissue N Vert (N Perp) to ST Pogonion (mm)	Distance between Soft Pogonion (Pg') and perpendicular line to the palatal plane via the point Soft Nasion (N')
	Sn'-Me' (mm)	Distance between Subnasale (Sn) and soft Menton (Me')
**SOFT TISSUE PROPORTIONS**	g'-sn'/sn'-me' (%)	Ratio between Glabela (G') - Subnasale (Sn) line and Subnasale (Sn) - soft Menton (Me') line
	g'-sn'/sn'-gn' (%)	Ratio between Glabela (G') - Subnasale (Sn) line and Subnasale (Sn) - soft Gnation (Gn') line
	Sn-Stomion / Sn-Me (%)	Ratio between Subnasale (Sn) - Stomion line and Subnasale (Sn) - soft Menton (Me') line
**AIR WAYS ANGULAR**	OPT - NS (º)	Angle formed between Odontoid line (OPT) and Nasion (N') - Sella (S) line
**AIR WAY LINEAR**	Lower Airway: Oro-pharyngeal	Distance between the point on the back contour of the soft palate and the nearest point on the pharyngeal wall.
	Upper Airway: Naso-pharyngeal	Distance between the point at the intersection of the rear edge of the tongue with the lower edge of the jaw and the nearest point of the rear pharyngeal wall.
	Anterior nasal cavity height (mm)	Distance between the SNP and the nearest adenoid tissue
	Posterior nasal cavity height (mm)	Shortest distance between the base of the tongue and the posterior wall of the pharynx
	H - PP (ANS-PNS) (mm)	Distance between Hyoid bone (H) and palatal plane
	PNS to Basion (mm)	Distance between Posterior Nasal Spine (PNS) and Basion

Supplementary Table 3. Supplementary craniometric mean values defined in each skeletal cluster.

	Cephalometric supplementary measurements	Global		C1 (n=21)		C2 (n=40)		C3 (n=70)		C4 (n=8)		C5 (n=34)		C6 (n=39)	
		Mean	DS	Mean	DS	Mean	DS	Mean	DS	Mean	DS	Mean	DS	Mean	DS
DENTAL ANGULAR	Interincisal Angle (U1-L1) (º)	136.53	11.48	133.99	9.97	136.20	10.59	134.57	11.10	143.28	10.97	135.42	9.84	141.28	13.19
	U1 - NA (º)	25.65	6.49	26.84	5.90	23.65	6.82	26.12	7.20	26.10	4.87	26.72	4.39	25.21	6.39
	U1 - SN (º)	105.79	7.55	106.58	7.25	100.43	8.04	106.17	7.31	106.64	5.82	109.66	4.88	106.69	6.89
	U1 - Palatal Plane (º)	114.61	7.15	113.44	6.99	111.89	7.26	114.61	7.25	116.10	4.52	116.75	5.05	115.85	7.89
	U1 - FH (º)	116.56	6.98	116.85	6.81	112.30	7.08	116.85	7.29	119.26	3.95	118.91	4.92	117.63	6.51
	L1 - NB (º)	19.18	7.19	20.32	5.67	20.86	5.59	20.38	6.36	15.64	10.16	19.56	7.26	15.12	8.13
	L1 to A-Pg (º)	23.48	5.94	25.02	5.41	23.19	4.86	23.82	5.39	23.54	9.47	24.54	5.78	21.44	6.71
	L1 - FH (º)	73.09	8.03	70.83	6.41	68.50	5.51	71.43	6.52	82.55	10.32	74.34	7.11	78.92	8.45
	IMPA (L1-MP) (º)	86.90	7.88	84.63	6.27	84.94	7.69	88.17	7.47	75.80	9.48	89.77	6.14	87.55	7.86
	L6 long axis - MP (º)	82.09	8.79	79.29	5.86	79.40	8.22	83.44	9.50	77.04	3.56	82.06	5.77	84.96	10.46
DENTAL LINEAR	Overjet (mm)	1.14	2.27	1.02	1.82	0.81	2.37	1.38	1.89	-2.60	2.75	1.24	2.08	1.80	2.24
	Overbite (mm)	1.33	1.73	0.34	1.77	1.17	1.50	1.18	1.57	0.64	1.46	1.28	1.44	2.45	1.93
	U1 - NA (mm)	5.90	2.22	7.14	1.98	5.55	2.20	6.05	2.44	6.43	2.36	6.30	1.66	4.92	1.82
	U1 to Occlusal Plane (mm)	0.30	1.64	-0.02	1.86	0.15	1.62	0.17	1.52	0.19	1.38	0.32	1.69	0.87	1.62
	U1 - PP (UADH) (mm)	27.69	3.35	32.69	2.27	29.22	3.22	27.10	2.18	26.68	1.86	28.18	2.38	24.41	2.50
	U1 to Nasion Perp (mm)	7.09	4.47	7.22	4.22	3.53	4.50	7.02	4.10	10.68	3.85	9.39	3.51	8.09	3.61
	L1 - NB (mm)	2.97	2.37	4.33	2.49	3.82	1.80	3.33	2.01	2.35	2.79	2.69	2.22	1.11	2.30
	L1 Protrusion (L1-APg) (mm)	2.88	2.69	3.68	2.60	3.76	1.85	3.21	2.53	4.25	2.99	2.68	2.61	0.85	2.63
	L1 to Occlusal Plane (mm)	1.00	1.45	0.37	1.48	1.00	1.63	0.97	1.28	0.39	1.71	0.96	1.46	1.54	1.26
	L1 - MP (LADH) (mm)	39.74	3.66	44.85	3.44	40.65	2.58	38.33	2.56	35.79	3.40	42.28	2.70	37.33	2.59
	L1 Tip - VRP (mm)	68.61	7.30	72.11	7.58	64.15	6.55	65.90	5.06	69.84	8.18	77.54	4.78	68.20	5.08
	U6 - PT Vertical (mm)	17.18	3.82	18.81	4.58	13.96	3.57	16.46	2.76	17.64	3.16	20.49	2.94	17.94	2.99
	U6 - PP (UPDH) (mm)	23.87	2.54	27.52	1.72	24.12	2.32	23.21	1.76	22.64	1.38	25.38	1.71	21.89	2.34
	L6 - MP (LPDH) (mm)	31.82	3.34	36.23	2.82	31.57	2.53	30.53	2.46	28.25	2.24	34.62	2.83	30.40	2.48
	Molar Relation (mm)	-4.66	2.26	-5.30	2.09	-4.83	1.94	-4.36	2.16	-9.29	1.77	-4.48	1.76	-3.93	2.12
SOFT TISSUE ANGULAR	NLA (Nasal Angle) (º)	131.98	8.75	128.92	7.56	134.64	8.99	133.17	8.45	130.50	8.82	129.34	7.71	131.28	9.16
	Facial Convexity (G'-Sn-Pg') (º)	131.32	113.00	172.71	3.78	153.00	75.89	143.17	98.43	0.62	173.76	111.13	133.57	111.02	134.70
	H-Angle (Pg'UL-Pg'N') (º)	8.06	4.38	7.91	2.63	9.01	3.74	8.23	3.98	3.93	5.24	8.37	5.17	7.41	4.87
SOFT TISSUE LINEAR	Upper Lip - S Line (mm)	-3.54	2.30	-4.72	1.74	-3.51	2.05	-3.26	2.27	-4.23	2.45	-3.21	2.61	-3.64	2.30
	Upper Lip - VRP (mm)	82.19	7.95	85.94	7.25	77.59	7.28	78.70	5.26	78.91	7.71	92.23	5.72	83.17	5.38
	STissue N Vert (N Perp) to Upper Lip (mm)	13.76	4.65	14.52	5.19	10.71	4.29	12.75	3.78	16.58	4.70	16.39	4.25	15.47	4.03
	Lower Lip - S Line (mm)	-1.47	2.32	-1.94	1.93	-0.77	2.20	-1.14	2.13	-0.73	3.03	-1.56	2.42	-2.63	2.22
	Lower Lip to E-Plane (mm)	-4.18	2.63	-5.02	2.14	-3.39	2.54	-3.76	2.40	-3.54	3.42	-4.35	2.87	-5.30	2.43
	Lower Lip - VRP (mm)	81.86	8.16	85.83	7.89	76.41	7.25	78.41	5.36	81.81	8.61	92.24	5.14	82.55	5.48
	STissue N Vert (N Perp) to Lower Lip (mm)	14.33	4.88	15.32	6.01	10.79	4.46	13.30	3.83	20.70	5.36	16.96	3.82	15.68	3.69
	STissue N Vert (N Perp) to ST Pogonion (mm)	13.03	6.30	13.26	7.58	6.82	5.33	11.55	4.35	22.55	4.30	16.61	4.15	16.88	3.89
	Sn'-Me' (mm)	69.87	6.75	81.36	6.91	72.23	5.06	66.95	4.21	66.88	3.68	72.97	3.78	64.69	4.80
SOFT TISSUE PROPORTIONS	g'-sn'/sn'-me' (%)	95.30	8.21	89.39	6.14	94.24	7.98	96.63	7.50	93.83	7.05	92.41	8.06	99.88	7.81
	g'-sn'/sn'-gn' (%)	105.77	9.37	99.37	7.41	103.99	8.37	106.80	8.37	102.51	7.81	103.09	9.47	112.02	9.21
	Sn-Stomion / Sn-Me (%)	33.40	2.73	32.83	2.83	33.58	2.39	33.47	2.66	32.40	1.63	33.88	2.79	33.18	3.08
AIR WAYS ANGULAR	OPT - NS (º)	99.72	17.83	102.73	7.02	99.72	37.71	101.25	8.21	97.71	5.07	96.46	7.59	98.66	6.36
AIR WAY LINEAR	Lower Airway: Oro-pharyngeal	11.28	5.15	12.10	3.55	11.58	6.26	9.81	3.16	9.83	2.69	12.68	6.02	12.28	6.21
	Upper Airway: Naso-pharyngeal	14.68	5.43	13.99	2.61	15.19	6.86	13.60	2.78	15.11	1.81	16.49	6.80	14.77	6.90
	Anterior nasal cavity height (mm)	49.23	5.07	55.97	4.01	51.28	3.77	47.08	3.49	45.31	1.94	51.55	3.61	46.30	5.47
	Posterior nasal cavity height (mm)	80.55	6.58	89.68	4.44	83.15	4.54	77.65	3.46	76.04	1.78	82.26	8.74	77.82	5.94
	H - PP (ANS-PNS) (mm)	-60.29	8.59	-69.62	6.88	-59.87	8.31	-57.25	7.19	-54.50	3.58	-66.33	7.20	-57.32	7.37
	PNS to Basion (mm)	43.67	4.15	44.83	4.11	43.04	3.38	41.83	3.38	41.45	3.44	47.73	4.34	43.95	3.31
